# Conjugated equine estrogen and medroxyprogesterone acetate are associated with decreased risk of breast cancer relative to bioidentical hormone therapy and controls

**DOI:** 10.1371/journal.pone.0197064

**Published:** 2018-05-16

**Authors:** Zexian Zeng, Xia Jiang, Xiaoyu Li, Alan Wells, Yuan Luo, Richard Neapolitan

**Affiliations:** 1 Department of Preventive Medicine, Northwestern University Feinberg School of Medicine, Chicago, Illinois, United States of America; 2 Department of Biomedical Informatics, University of Pittsburgh, Pittsburgh, Pennsylvania, United States of America; 3 Department of Social and Behavioral Sciences, Harvard T.H. Chan School of Public Health, Boston, Massachusetts, United States of America; 4 Department of Pathology, University of Pittsburgh and Pittsburgh VA Health System, Pittsburgh, Pennsylvania, United States of America; Turun Yliopisto, FINLAND

## Abstract

**Objective:**

By the 1990s it became popular for women to use hormone therapy (HT) to ease menopause symptoms. Bioidentical estrogen and progesterone are supplements whose molecular structures are identical to what is made in the human body, while synthetic supplements are ones whose structures are not. After the Women’s Health Initiative found that the combined use of the synthetics conjugated equine estrogen (CEE) and medroxyprogesterone acetate (MPA) increased breast cancer risk, prescriptions for synthetic HT declined considerably. Since then there has been an increased interest in bioidentical HT; today there are a plethora of websites touting their benefits. However, no peer-reviewed articles support these claims. We performed a retrospective study with the objective of verifying the *hypothesis that bioidentical HT is associated with decreased breast cancer risk than CEE & MPA*.

**Methods:**

We searched The Northwestern Medicine Enterprise Data Warehouse for women who initiated HT use after age 50. Women who did not take any HT drug after age 50 served as controls. Nine HT protocols were investigated for breast cancer risk.

**Results:**

Significant results include *CEE Alone* is associated with decreased breast cancer risk (HR = 0.31), *Other Synthetic Estrogen Alone* is associated with increased breast cancer risk (HR = 1.49), *Bioidentical Estrogen Alone* is associated with decreased breast cancer risk(HR = 0.65), *CEE & MPA* is associated with reduced breast cancer risk (HR = 0.43), and *CEE & MPA* is associated with reduced breast cancer risk relative to *Bioidentical Estrogen & Progesterone* (HR = 0.25).

**Discussion:**

Our results indicate *CEE & MPA* is superior to bioidentical HT as far as breast cancer risk. Furthermore, this combination is associated with decrease of breast cancer risk, contrary to previous findings. Additional retrospective studies are needed to confirm our results.

## Introduction

By the 1990s it became popular for women to use hormone therapy (HT) to ease symptoms of menopause such as hot flashes and night sweats, which persist in more than half of women for more than seven years [1]. Menopausal women less than 60 years of age or less than 10 years past menopause, with bothersome vasomotor symptoms (VMS), without contraindications, excess cardiovascular, or excess breast cancer risks, and are willing to take menopausal HT, are often advised to initiate HT [2]. HT remains the most effective way to ameliorate VMS and the genitourinary syndrome of menopause (GSM) [3]. Extended use of HT after menopause is considered to have other health and quality of life benefits [4]. Bioidentical estrogen and progesterone are HTs whose molecular structures are identical to what is made in the human body, while synthetic estrogen and progesterone are ones whose molecular structures are not. A woman may take estrogen alone, progesterone alone, or both.

To address the risks associated with estrogen, the Women’s Health Initiative (WHI)-Estrogen-Alone Trial was initiated in 1992 [5–7]. This study was a *case-control* study that investigated the effect of the popular synthetic conjugated equine estrogens (CEE). This study investigated the effect of chronic disease incidence among postmenopausal U.S. women with a prior hysterectomy. Over 5,000 women in the HT group took a daily dose of CEE for an average of about 6 years. The researchers followed them for several years, and their outcomes were compared to more than 5,000 in the control group who took no HT. With a p-value of 0.02 the study indicated that CEE had a protective effect against breast cancer. Relative to controls the Hazard Ratio was 0.77 (0.62, 0.95). Anderson et al. [8] also reported a long-term reduced breast cancer risks in CEE-only users based on an analysis of the WHI data. The WHI study additionally investigated the combined use of CEE with the synthetic progesterone medroxyprogesterone acetate (MPA). With a p-value of 0.004 relative to controls the Hazard Ratio concerning breast cancer risk for the HT group was 1.25 (1.07, 1.46) [9].

The Million Women Study (MWS) [10] was a *prospective* HT study that followed 1,084,110 UK women aged 50–64 years between 1996 and 2001. Of those 48,386 took equine estrogen only, and 56,322 took estradiol. With a p-value < 0.0001 relative to controls the Hazard Ratio concerning breast cancer risk for the equine oestrogen group was 1.29 (1.16, 1.43), and the Hazard Ratio concerning breast cancer risk for the estradiol group was 1.24 (1.12, 1.37). The MWS results are similar to estimates derived from a collaborative reanalysis of 51 epidemiological studies in 1997 [11]. The MWS investigated the combined use of CEE with the synthetic progesterone medroxyprogesterone acetate (MPA). With p-value < 0.0001 the Hazard Ratio concerning breast cancer risk for the HT group was 1.62 (1.34, 1.96) for total duration < 5 years, and 2.42 (2.08, 2.81) for total duration > 5 years (9).

Lyytinen et al. [12] did a *retrospective* HT study analyzing Finnish women older than age 50 years using oral or transdermal estradiol (n = 84,729), oral estriol (n = 7,941), or vaginal estrogens (n = 18,314) for at least 6 months during 1994–2001. They were followed for breast cancer with the aid of the Finnish Cancer Registry to the end of 2002. The standardized incidence ratio for breast cancer, when estradiol was used for less than 5 years, was 0.93 (0.80, 1.04), and, when estradiol was used for 5 years or more, was 1.44 (1.29, 1.59).

Lyytinen et al. [13] also did a *retrospective* HT study investigating Finnish women who used combined estradiol-progestogen therapy. The standardized incidence ratio for breast cancer with estradiol-progestogen for women who used the therapy for 3–5 years was 1.31(1.20, 1.42), and for women who used it for 10 or more years was 2.07(1.84, 2.30).

A difficulty with the MWS study and the Finnish studies is that they grouped all estradiol medications. Yet some are bioidentical and some are synthetic. For example, estradiol acetate is bioidentical and ethinyl estradiol is synthetic. The most notable study explicitly investigating bioidentical HT is the E3N-EPIC study, which is a French *prospective* study investigating cancer risk factors in 98,997 women born between 1925 and 1950 [14]. Very few women in the study took CEE or other synthetic estrogens. The significant results are as follows. The Hazard Ratio for breast cancer risk for women taking bioidentical estrogen alone was 1.1 (0.8, 1.6); the Hazard Ratio for breast cancer risk for women taking bioidentical estrogen and synthetic progesterone was 1.4 (1.2, 1.7); and the Hazard Ratio for breast cancer risk for women taking bioidentical estrogen and bioidentical progesterone was 0.9 (0.7, 1.2).

Following the publication of the results of the combined CEE and MPA investigation in the WHI study, prescriptions for this combination of drugs declined considerably in the USA [15]. Since then there has been an increased interest in bioidentical HT, and today there are a plethora of websites touting the benefits of bioidentical HT. Files et al. [16] state “Our research on resources available to patients yielded more than 70 books listed on Amazon.com, numerous blogs and Web sites, online pharmacies, and an internet company endorsing affiliated physicians as experts in the use of compounded bioidentical hormone therapy (CBHT).” It is estimated that bioidentical hormone therapy is a multibillion-dollar industry, affecting millions of women [15,16]. Besides the argument that bioidentical hormones are “natural”, the reasons often given for making the bioidentical choice are as follows. First, synthetic HT can be deleterious because studies indicate synthetic estrogen and progesterone combined is associated with the increased risk of breast cancer. Second, bioidentical HT may not be deleterious because studies indicate combined estrogen and progesterone (bioidentical) is slightly associated with decreased breast cancer. However, this last claim is based on the E3N-EPIC study, and the 95% interval for the breast cancer Hazard Ratio in that study was (0.7, 1.2). Furthermore, there were only 55 breast cancer occurrences in the cohort in that study. So, the results do not preclude the possibility that combined biosynthetic HT is associated with increased breast cancer risk.

Santen et al. [17] state “Despite the contention by proponents that CBHT has been found to be safer, more efficacious, or less likely to cause breast or uterine cancer than FDA-approved HT, no reports published in peer-reviewed journals support this claim.” Further studies are necessary concerning the use of bioidentical HT. However, case-control and prospective studies are costly and time consuming, which means the results of a large-scale study involving bioidentical HT may not be on the horizon. We are in the era of big data, and have abundant electronic health record (EHR) data. To further investigate all HT therapy, we mined 12,404 HT cases and 27,642 controls from the Northwestern Medicine Medical Data Warehouse (NMEDW). In a retrospective study, we investigated the breast cancer risk for various HT protocols. *Our hypothesis was that bioidentical HT is associated with less breast cancer risk than CEE & MPA and other synthetic HT*.

## Methods

### Data mining methods

The NMEDW is a joint initiative across the Northwestern University Feinberg School of Medicine and Northwestern Memorial HealthCare. The NMEDW was used to search for data. To approximately include only women who took hormone for the purpose of HT, only women older than age 50 were included. For these women, all drugs taken by them after age 50 were retrieved. Women who took any HT drug after age 50 were used as cases; women who did not take any type of HT drug after age 50 were used as controls. After obtaining the study cohorts and drugs, breast cancer events were obtained from ICD9 codes from the database. The earliest date of HT usage was a woman taking MPA on 1/1/1983.

For each patient, the last event of immunization, treatment, procedure, consultation, outpatient service, nurse visit, or office visit was recorded as the last contact date. For the women who did not develop breast cancer before the last contact date, the time to censoring was the number of days from the women’s 50th birthday to the last contact date. For the women who developed breast cancer before the last contact date, the time to event was the number of days from the 50th birthday to the breast cancer diagnosis date.

There are many types of HT drugs available. Accounting for so many different types of drugs in the study would impair the study power. To solve this problem, the HT drug names were grouped into 8 categories. The names of drugs included in each category are as follows. **CEE**: Conjugated equine estrogen, Premarin; **Other Synthetic Estrogen**: Cenestin, Enjuvia, Menest, Esterified Estrogen, OrthoEst, Estropipate, Piperazine Estrone Sulfate, Ethinyl Estradiol, Ogen, Estinyl, Ethinyloestradiol, Ethinylestradiol; **MPA**: Medroxyprogesterone acetate, Amen, Cycrin, Provera, MPA, Depo-Provera; **Other Synthetic Progesterone**:, Norgestrel, Norethindrone, Ortho-Micronor, Micronor, Nor-QD, Norethindrone acetate, Aygestin, cyproterone acetate, CPA, Androcur, Retroprogesterone, Trengestone, Reteroid, Retroid, Retrone, Nomegestrol Acetate, Promegestone, Chlormadinone Acetate, CMA, Levonorgestrel, Drospirenone, Desogestrel, Norgestimate; **Combined CEE and MPA**: Premphase, Prempro; **Other Combined Synthetics**: Femhrt, Diane, Diane-35, Alesse, Desogestrel and Ethinyl Estradiol, Desogen, Norethindrone and Ethinyl Estradiol, Jevantique, Jinteli, Ethinyl Estradiol & Levonorgestrel, Levlen, Levlite, Lybrel, Levora, Nelova, Ortho-Cyclen, Ovral, Preven EC, Sronyx, Tri-Levlen, Norgestrel-Ethinyl Estradiol, Ortho-Cyclen, Ortho Tri-Cyclen, Previfem, Sprintec, Ortho Evra, Evra; **Bioidentical Estrogen**: Estradiol Oral, Estrace Oral, 17 Beta Estradiol, Micronized Estrogen, Estradiol Acetate Oral, Estradiol Hemihydrate Oral, Gynodiol Oral, Femtrace, Innofem, Estradiol Acetate Oral, Delestrogen, Estradiol Valerate, Estradiol Cypionate, Depo-Estradiol, Estradiol Transdermal, Alora, Climara, Esclim, Estraderm, Vivelle, Vivelle-Dot, Dot, Estrogel, Estrasorb, Estradiol Transdermal, Transdermal Estradiol, FemPatch, Menostar, Elestrin, Divigel, Evamist, Estrasorb, Minivelle; **Bioidentical Progesterone**: Micronized Progesterone, Prometrium, Prochieve, Crinone. We searched for records containing any of these drugs. Any record whose drug name or decription contained the word ‘cream’ or ‘vaginal’ was discarded.

Women were assigned to the following 9 HT protocols:

1. CEE Alone
2. MPA Alone
3. Other Synthetic Estrogen Alone
4. Other Synthetic Progesterone Alone
5. Bioidentical Estrogen Alone
6. Bioidentical Progesterone Alone
7. CEE and MPA
8. Other Synthetic Estrogen and Synthetic Progesterone
9. Bioidentical Estrogen and Bioidentical Progesterone

Some women followed more than one HT protocol. Such women were assigned to the protocol of largest duration.

Drug dosage was often not available. Furthermore, even if we could stratify the data by usage, there would likely not be enough users in any particular usage category to reach statistical significance. So, we have made the assumption that each of the 9 categories has a similar distribution of drug usage, and so our comparison is a comparison of HT protocols ranging over similar distributions. This is one of several weaknesses of a retrospective study relative to a case-control study or prospective study. However, a big advantage of a retrospective study is that is much less costly in terms of dollars and time. This matter is discussed more in the Discussion Section.

### Statistical methods

Using Cox regression, hazard ratios, were computed for each of the HT protocols relative to the control group. Furthermore, *p*-values were computed using the one-sided log-rank test, where the null hypothesis is that the area under the survival curve for the control group is smaller than or equal to the area under the survival curve for the HT group. Additional studies were performed comparing HT protocols, and hazard ratios and *p*-values were computed in the same manner.

### Ethics statement

This paper is a result of the PROTOCOL TITLE: A New Generation Clinical Decision Support System, which was approved by Northwestern University IRB #: STU00200923-MOD0006.

## Results

**Fig 1:**
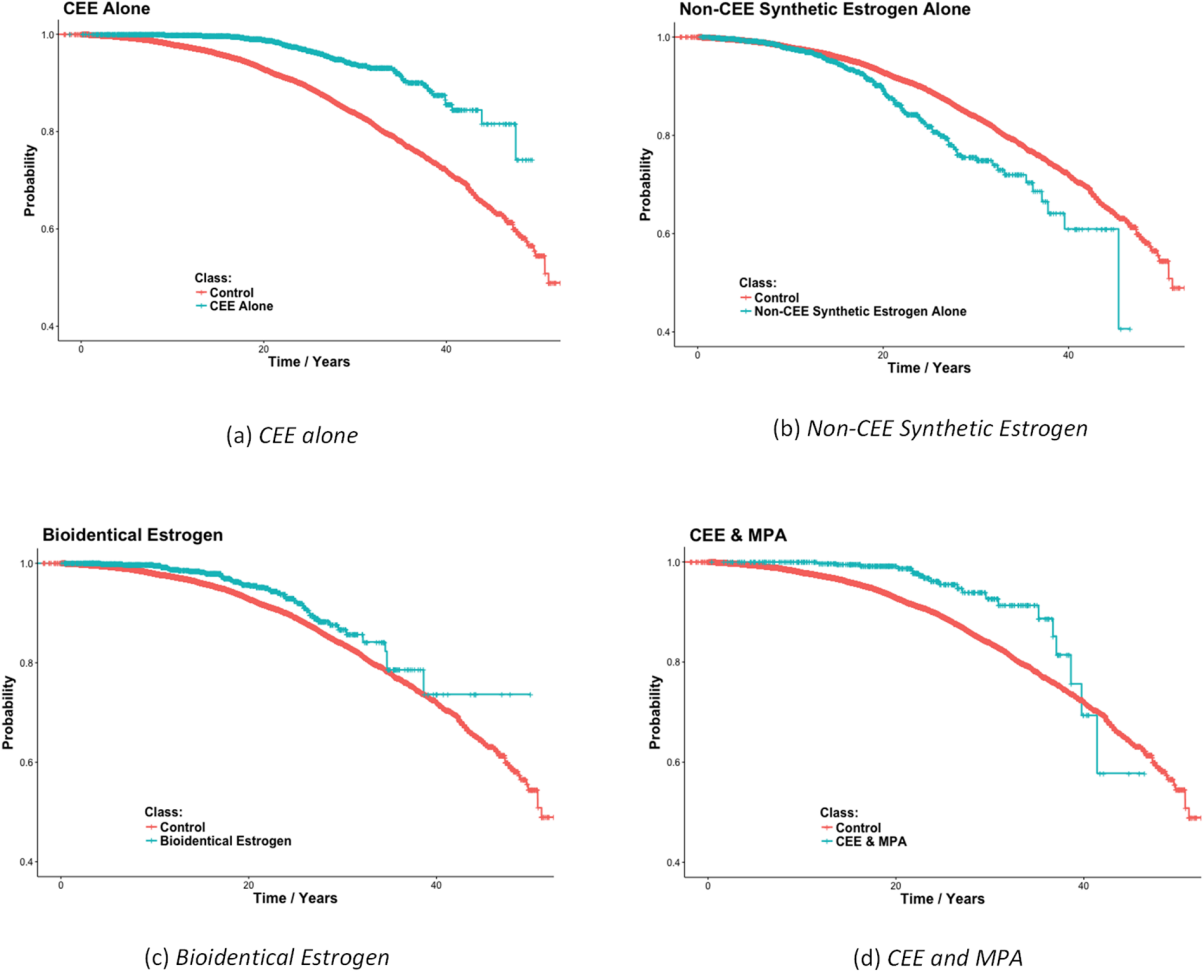
Kaplan-Meier plots for the 4 HT protocols found to significantly affect breast cancer in Table 1. Each graph also shows the plot for the control group consisting of women who did not take HT. The plots show the probability of surviving (not getting breast cancer) up to the amount of time after age 50.

In this study, we mined 12,404 HT cases and 27,642 controls. The mean follow-up time for cases and controls are 15.4 (SD = 9.7) years and 17.8 (SD = 11.1) years. Table 1 shows the results of comparing the 9 HT protocols to the controls who used no HT. Both *CEE Alone*, and *Bioidentical Estrogen Alone* provided a significant protective effect against breast cancer. The first result is consistent with the results of the WHI study. *Other Synthetic Estrogens Alone* is significantly associated with increased risk of breast cancer. Finally, *CEE & MPA* together is also significantly associated with decreased breast cancer risk. *Bioidentical Progesterone Alone* and combined *Bioidentical Estrogen & Bioidentical Progesterone* were both shown to be associated with increased risk of breast cancer, but the results were not significant. Fig 1 shows Kaplan-Meier plots for the four significant findings. The plots show the probability of surviving (not getting breast cancer) up to the amount of time after age 50.

**Table 1:** Hazard ratios for 9 HT protocols relative to a control group consisting of women who did not take any HT, 95% confidence intervals for the hazard ratios, and *p*-values obtained using the one-sided log-rank test, where the null hypothesis is that the area under the survival curve for the control group is smaller than or equal to the area under the survival curve for the HT group.

HT Protocol	HR (95% CI)	*p*-Value	# Cases	# BC Cases (%)
*CEE Alone*	0.31 (0.25, 0.38)	**1.00×10**^**−16**^	2556	85 (3.33%)
*MPA Alone*	0.85 (0.50, 1.54)	0.28	442	14 (3.17%)
*Other Synthetic Estrogen Alone*	1.49 (1.25, 1.78)	**3.48×10**^**−6**^	1349	133 (9.86%)
*Other Synthetic Progesterone Alone*	0.64 (0.27, 1.56)	0.17	417	5 (1.12%)
*Bioidentical Estrogen Alone*	0.65 (0.49, 0.85)	**0.00076**	1441	55 (3.82%)
*Bioidentical Progesterone Alone*	1.18 (0.69, 2.04)	0.274	265	13 (4.91%)
*CEE & MPA*	0.43 (0.28, 0.67)	**0.000087**	453	20 (4.42%)
*Other Synth*. *Estr*. *& Synth*. *Prog*.	1.03 (0.84, 1.27)	0.38	2512	96 (3.82%)
*Bioid*. *Estrogen & Bioid*. *Prog*.	1.05 (0.59, 1.85)	0.438	288	12 (4.17%)

The number of controls was 27,642, and the number of controls realizing breast cancer was 2,399.

**Fig 2:**
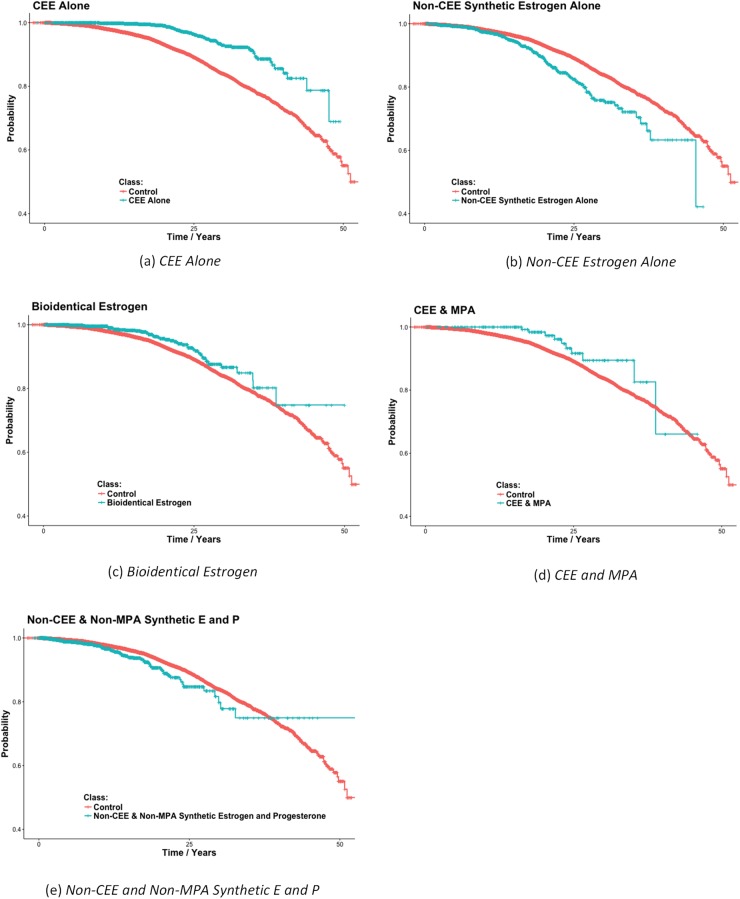
Kaplan-Meier plots for the 5 HT protocols found to significantly affect breast cancer in Table 2. Each graph also shows the plot for the control group consisting of women who did not take HT. Only women initiating drug usage after 01/01/2004 were included in the studies producing these results. The plots show the probability of surviving (not getting breast cancer) up to the amount of time after age 50.

The results in Table 1 indicate *CEE & MPA* is associated with less breast cancer risk than both other synthetics and bioidentical hormones. However, our data dates back to 1983, and bioidentical HT did not see substantial usage until after the results of the WHI appeared in the early part of the 21^st^ century. Breast cancer diagnosis had improved by this time; the possibility exists that the population of women on bioidentical HT is skewed towards receiving better diagnostics, making breast cancer diagnoses appear more frequently in this group, even if they are not more likely to get breast cancer. To account for this possibility, we repeated the study using only women (cases and controls) who were first prescribed any medication after 01/01/2004. Table 2 shows the results of this study. These results mirror those obtained for all women, except there is one additional significant result, namely that the combined usage of *Other Synthetic Estrogen & Synthetic Progesterone* is associated with increased risk of breast cancer. Fig 2 shows Kaplan-Meier plots for the 5 significant findings.

**Table 2:** Hazard ratios for 9 HT groups relative to a control group consisting of women who did not take any HT, 95% confidence intervals for the hazard ratios, and *p*-values obtained using the one-sided log-rank test, where the null hypothesis is that the area under the survival curve for the control group is smaller than or equal to the area under the survival curve for the HT group.

HT Protocol	HR (95% CI)	*p*-Value	# Cases	# BC Cases (%)
*CEE Alone*	0.34 (0.27, 0.44)	**1.00×10**^**−16**^	2024	68 (3.36%)
*MPA Alone*	1.07 (0.64, 1.78)	0.40	425	14 (3.17%)
*Other Synthetic Estrogen Alone*	1.55 (1.30, 1.86)	**5.80×10**^**−7**^	1314	130 (9.89%)
*Other Synthetic Progesterone Alone*	0.84 (0.38, 1.87)	0.33	416	5 (1.20%)
*Bioidentical Estrogen Alone*	0.66 (0.50, 0.88)	**0.0023**	1350	49 (3.63%)
*Bioidentical Progesterone Alone*	1.23 (0.71, 2.12)	0.23	265	13 (4.91%)
*CEE & MPA*	0.56 (0.30, 1.03)	**0.03**	200	10 (5.00%)
*Other Synth*. *Estr*. *& Synth*. *Prog*.	1.38 (1.10, 1.72)	**0.002**	2222	82 (3.69%)
*Bioid*. *Estrogen & Bioid*. *Prog*.	1.12 (0.63, 1.97)	0.35	282	12 (4.17%)

Only women initiating drug usage after 01/01/2004 are included. The number of controls was 24,301, and the number of controls realizing breast cancer was 2016.

Of interest is the effect on breast cancer of long-term usage of HT. Tables 3 and 4 show the same results as in Tables 1 and 2, but including only cases that used HT at least 5 years. We see from these tables that the results concerning the protective effect of *CEE Alone* remain significant for all data and for data after 01/01/2004, when we consider only cases that used HT at least 5 years. Furthermore, the results indicate that the protective effect increases with longer use. The results concerning *Other Estrogen Alone* again indicate that it is associated with increased risk, but the results are not significant. The results concerning *Bioidentical Estrogen Alone* remain that it is associated with decreased risk both based on all data and based on data after/01/01/2004, but the results are significant only for all the data. The results concerning combined usage of *CEE & MPA* remain that it is associated with decreased risk both based on all data and based on data after/01/01/2004, but the results are significant only for all the data. One oddity is that the results for combined *Other Synthetic Estrogen & Progesterone* reverse the association from increasing risk to decreasing risk. However, these results are not significant.

**Table 3:** Hazard ratios for 9 HT protocols, consisting of women who took HT at least 5 years, relative to a control group consisting of women who did not take any HT, 95% confidence intervals for the hazard ratios, and *p*-values obtained using the one-sided log-rank test, where the null hypothesis is that the area under the survival curve for the control group is smaller than or equal to the area under the survival curve for the HT group.

HT Protocol	HR (95% CI)	*p*-Value	# Cases	# BC Cases (%)
*CEE Alone*	0.28 (0.19, 0.44)	**4.48×10**^**−9**^	604	23 (3.48%)
*MPA Alone*	NA	NA	NA	NA
*Other Synthetic Estrogen Alone*	1.45 (0.78, 2.7)	0.12	86	10 (11.62%)
*Other Synthetic Progesterone Alone*	NA	NA	NA	NA
*Bioidentical Estrogen Alone*	0.69 (0.39, 1.21)	0.082	202	12 (5.94%)
*Bioidentical Progesterone Alone*	0.88 (0.12, 6.26)	0.45	20	1 (5%)
*CEE & MPA*	0.47 (0.78, 2.7)	**0.009**	194	10 (5.15%)
*Other Synth*. *Estr*. *& Synth*. *Prog*.	0.66 (0.40, 1.10)	**0.055**	305	15 (4.92%)
*Bioid*. *Estrogen & Bioid*. *Prog*.	1.43 (0.54, 3.81)	0.24	51	4 (7.84%)

The number of controls was 27,642, and the number of controls realizing breast cancer was 2399.

**Table 4:** Hazard ratios for 9 HT protocols, consisting of women who took HT at least 5 years, relative to a control group consisting of women who did not take any HT, 95% confidence intervals for the hazard ratios, and *p*-values obtained using the one-sided log-rank test, where the null hypothesis is that the area under the survival curve for the control group is smaller than or equal to the area under the survival curve for the HT group.

HT Protocol	HR (95% CI)	*p*-Value	# Cases	# BC Cases (%)
*CEE Alone*	0.28 (0.16, 0.49)	**4.19×10**^**−6**^	415	12 (2.89%)
*MPA Alone*	NA	NA	NA	NA
*Other Synthetic Estrogen Alone*	1.44 (0.72, 2.9)	0.15	72	8 (11.11%)
*Other Synthetic Progesterone Alone*	NA	NA	NA	NA
*Bioidentical Estrogen Alone*	0.72 (0.37, 1.39)	0.16	156	9 (5.77%)
*Bioidentical Progesterone Alone*	1.05 (0.15, 7.48)	0.48	19	1 (5.26%)
*CEE & MPA*	0.75 (0.31, 1.8)	0.26	78	5 (6.41%)
*Other Synth*. *Estr*. *& Synth*. *Prog*.	0.83 (0.44, 1.5)	0.27	225	10 (4.44%)
*Bioid*. *Estrogen & Bioid*. *Prog*.	1.46 (0.54, 3.81)	0.32	51	4 (7.84%)

Only women initiating drug usage after 01/01/2004 are included. The number of controls was 24,301, and the number of controls realizing breast cancer was 2016.

**Fig 3:**
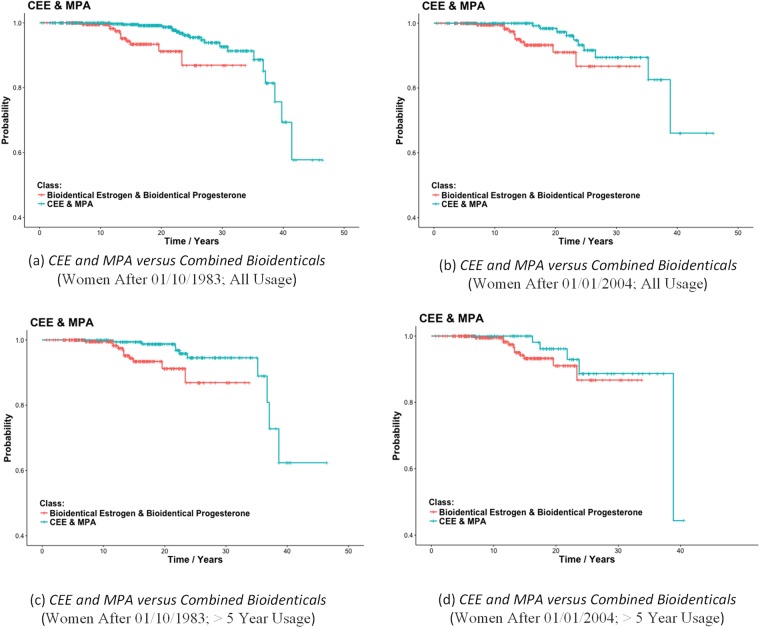
Kaplan-Meier plots for *CEE & MPA* users versus *Bioidentical Estrogen & Bioidentical Progesterone* users. The plots show the probability of surviving (not getting breast cancer) up to the amount of time after age 50.

We compared the combined use of *CEE & MPA* directly to the combined use of *Bioidentical Estrogen & Bioidentical Progesterone*. Table 5 shows the results. We see that *CEE & MPA* is significantly associated with less breast cancer risk than the bioidentical hormones, regardless of whether we include all women, or only women after 01/01/2004 and regardless of whether we consider all usage or only usage greater than 5 years. However, the results including only women after 01/01/2004 and with usage > 5 years are not significant. Fig 3 shows Kaplan-Meier plots for these comparisons.

**Table 5:** Hazard ratios for women who used *CEE & MPA* versus women who used *Bioidentical Estrogen & Bioidentical Progesterone*, 95% confidence intervals for the hazard ratios, and *p*-values obtained using the one-sided log-rank test, where the null hypothesis is that the area under the survival curve for the bioidentical users is smaller than or equal to the area under the survival curve for the *CEE & MPA* users.

Time Period	HR (95% CI)	*p*-Value
Women After 01/10/1983; All Usage	0.25 (0.12, 0.55)	**0.0002**
Women After 01/01/2004; All Usage	0.35 (0.14, 0.87)	**0.01**
Women After 01/10/1983; > 5 Year Usage	0.31 (0.13, 0.77)	**0.006**
Women After 01/01/2004; > 5 Year Usage	0.47 (0.15, 1.5)	0.10

## Discussion

We conducted a retrospective study investigating various HT protocols using 12,404 HT cases and 27,642 controls. We found *CEE Alone* to be the best estrogen alone therapy as far as breast cancer risk, and *CEE & MPA* together to be the best combined therapy. In fact, *Other Synthetic Estrogen Alone* was associated with an increased risk in breast cancer relative to non-HT users. *Bioidentical Estrogen Alone* was associated with a decreased risk of breast cancer, but not as much as *CEE Alone*. In a head-to-head comparison, the combined use of *CEE & MPA* was shown to be significantly associated with less breast cancer risk than combined bioidentical hormones.

The results of our study negated our hypothesis that *bioidentical HT is associated with less breast cancer risk than CEE & MPA*. However, it does support that *Bioidentical Estrogen Alone* is associated with less risk than *Other Synthetic Estrogen Alone*.

As far as bioidentical hormones, our results significantly support that a woman’s chances of breast cancer are greater on bioidentical HT than they are on *CEE & MPA* (p = 0.0002). Furthermore, the only bioidentical protocol that was shown to be significantly associated with decreased breast cancer risk was *Bioidentical Estrogen Alone* (p = 0.00076). Other bioidentical protocols were found to be associated with increased risk, but the results were not significant. However, we grouped all bioidentical estrogens and progesterone together. Anyone interested in forwarding the usage of a particular bioidentical HT protocol should conduct a study investigating that particular protocol before making further claims about its efficacy.

We found the combined use of *CEE & MPA* to be associated with reduced breast cancer risk, whereas the WHI initiative showed the opposite. There are numerous possible explanations for this discrepancy. First, the WHI study concerned women with a prior hysterectomy. Second, our study concerned only women visiting Northwestern Medical Complex. There could be life style factors in this population that affect the outcomes. Finally, our study includes many women who more recently took *CEE and MPA* than the WHI. Perhaps the composition of these drugs has changed in this century.

Our study was retrospective, and has all the weaknesses of retrospective, including not making drug dosage a variable. Nevertheless, retrospective data are increasingly available, making studies like ours possible without the cost and time involved in case-control studies and prospective studies. Retrospective studies can be highly supportive of a result if many such studies obtain the same results. So, to substantiate our results, we need additional retrospective studies. First, we need studies using other databases to see if our results are duplicated. Second, we can perform retrospective studies investigating how patient variables such as HER2 and ER status interact with *CEE & MPA* usage to affect breast cancer risk. For example, CEE *& MPA* might be protective only in ER positive women.

The question remains as to why CEE, with or without MPA, would be associated with reduced breast cancer. One possibility is as follows. Tamoxifen binds to estrogen receptors, but functions mainly as an antagonist, over-riding the agonist activity. CEE, which is a mixture of conjugated estrogens and steroid precursors, may function similarly with both agonist and antagonist elements. The differential effects mainly on bone and vascular tissue with estrogenic activities towards breast tissue argues for this dichotomous functionality.

There is a great deal of hype concerning the advantages of bioidentical hormone replacement therapy, with no real evidence indicated it is safer. In this study, we conducted a retrospective study showing that it is indeed not safer as far as breast cancer risk. One should be dubious of the unwarranted claims that bioidentical is safer, and that either case/control studies or more retrospective studies are needed before making any claims one way or the other.

## Supporting information

S1 Dataset The supporting file “S1_Dataset.zip” contains the datasets which were analyzed in the research described in this paper. The dataset label “Dataset1” contains all the data, and the dataset labeled “Dataset2” contains data only for these records pertaining to women who initiated drug usage after 01/01/2004.
(ZIP)
